# Tongkat Ali (*Eurycoma longifolia*): a possible therapeutic candidate against *Blastocystis* sp.

**DOI:** 10.1186/s13071-015-0942-y

**Published:** 2015-06-18

**Authors:** Sonal Girish, Suresh Kumar, Norhaniza Aminudin

**Affiliations:** Department of Parasitology, Faculty of Medicine, University of Malaya, 50603 Kuala Lumpur, Malaysia; Institute of Biological Sciences, Faculty of Science & University of Malaya Centre for Proteomics Research, Medical Biotechnology Laboratory, Faculty of Medicine, University of Malaya, 50603 Kuala Lumpur, Malaysia

**Keywords:** Allopathic drugs, Herbal extracts, Drug susceptibility assay, Subtypes, Blastocystis, *Eurycoma longifolia*

## Abstract

**Background:**

In the local Malaysian context, herbal plants such as *Eurycoma longifolia* (Tongkat Ali), *Orthosiphon stamineus* (MisaiKucing), *Ficus deltoidea* (Mas Cotek), *Zingiber officinale* (Halia Bara) and *Barringtonia racemosa* (Putat) are known and widely used for its therapeutic properties. The first part of this study aims to screen for the anti-protozoal activity of these herbal plant extracts against *Blastocystis* sp. isolate subtype (ST) 3. Herbal extract with the highest efficacy was further fractionized into water and ethyl acetate fractions and tested against ST1, ST3 and ST5 *Blastocystis* sp. isolates. These isolates were also exposed to allopathic drugs, Metronidazole (MTZ), Tinidazole, Trimethoprim-sulfamethoxazole(TMP-SMX), Ketoconazole and Nitazoxanide for comparison purpose.

**Methods:**

*Blastocystis* sp. isolates from human-derived stool samples were exposed to herbal extracts and allopathic drugs at a concentration of 0.1 mg/ml and 1.0 mg/ml and were incubated at 37 °C. Growth profile studies were carried out. After 72 h of treatment, the viability of *Blastocystis* sp. as a result of the effects of the drugs and herbal extracts were assessed.

**Results:**

Based on the screening process, amongst all the extracts, Tongkat Ali exhibited the highest anti-protozoal activity at 1.0 mg/ml. Between the water and ethyl acetate fractions of Tongkat Ali, the ethyl acetate fraction exhibited a slightly higher percentage of anti-protozoal activity at 1.0 mg/ml across subtypes, ST1 (94.9 %), ST3 (95.1 %) and ST5 (94.3 %). When tested with allopathic drugs, at the same concentration, MTZ exhibited the highest anti-protozoal activity across subtypes, ST1 (95.8 %), ST3 (93.4 %) and ST5 (90.8 %).

**Conclusion:**

This study is the first to describe the anti-protozoal properties of Tongkat Ali against *Blastocystis* sp. isolates*.* Ethyl acetate fraction of Tongkat Ali demonstrated the highest anti-protozoal activity against *Blastocystis* sp. isolates and showed a sizeable reduction in the cell count which was comparable with MTZ. Tongkat Ali also demonstrated a more uniformed sensitivity across subtypes in comparison to the allopathic drugs.

## Background

*Blastocystis* sp. recently classified as a Stramenopile [1], is a genetically diverse organism inhabiting the intestinal tract of a large range of host species including humans [2, 3]. There are up to about 17 subtypes within which subtype (ST) 1 to 9 are found in humans [4]. ST3 is known to be the predominant ST found in most human epidemiological studies [5]. Since its first description as early as 1900’s [6], despite an increasing number of studies, there still exists uncertainty and debate on its prevalence, pathogenicity, and clinical significance. This has resulted in the lack of availability of treatment and chemotherapeutic approaches against *Blastocystis sp*. A few antiprotozoal agents have been used against *Blastocystis sp.* infection, the most commonly used still being Metronidazole (MTZ), as the first-line treatment followed by Nitazoxanide, Trimethoprim-sulfamethoxazole (TMP-SMX), Ketoconazole, and Tinidazole as secondary treatments. Studies have shown that while MTZ demonstrates effectiveness in some individuals [7, 8] it has also shown to exhibit side effects and resistance in others [9, 10].

The present conflicting results in drug treatment forms the basis to explore alternative anti-protozoal agents and development of new therapeutic options focusing mainly on medicinal plants. The low cost, easy accessibility towards getting these plants, less side effects and a wider availability have made this option a more realistic alternative in recent times. Previously, a few traditional anti-diarrheic Thai medicinal herbs have been examined for its in-vitro activity against *Blastocystis hominis* which resulted in a non-comparable inhibitory activity to MTZ [11]. Another study tested *Blastocystis hominis* isolates from IBS patients mostly genotype-1 and demonstrated increased susceptibility to garlic at 0.01 mg/ml. Other investigational agents such as ginger, black pepper, and white cumin were tested but showed insignificant inhibitory effect against the parasite [12].

Herbs such as *Eurycoma longifolia* (Tongkat Ali), *Orthosiphon stamineus* (Misai Kucing), *Barringtonia racemosa* (Putat), *Zingiber officinale* (Halia Bara) and *Ficus deltoidea* (Mas Cotek) are amongst the most popular traditional folk medicines extensively used particularly in Malaysia, Indonesia, Thailand, Laos, Cambodia and Vietnam for its pharmacological properties. Tongkat Ali belongs to the family of Simaroubaceae and is mainly known for its aphrodisiac applications. Studies have shown that some of the compounds found in Tongkat Ali have been known to possess anti-bacterial [13], anti-tumoral [14], and anti-malarial properties [15]. Misai Kucing of the family Lamiaceae, has white and light purple flowers with the latter having higher quantity of the same bioactives. This plant possesses therapeutic properties that exhibit diuretic, antioxidant, anti-inflammatory, gastroprotective, antihypertensive, antidiabetic, antihyperlipidemic and antimicrobial activities [16]. The medicinal herb Putat, a tree in the family Lecythidaceae, is widely used for its anti-inflammatory and anti-cancer activity in Malaysia [17]. Secondary metabolites such as flavonoids, steroids, saponins and diterpenes have been found in this plant that contributes to its therapeutic activity. Based on previous studies, Putat not only exhibits antifungal properties, it also showed antibacterial activity [18]. Mas Cotek (family of Moraceae) is a plant that is traditionally used for post-delivery treatment, uterus contraction, treating gout, hypertension, diabetes, cholesterol and sugar reduction as well as for improving blood circulation [19]. This herb also possesses medicinal values involving the anti-oxidative activity and anti-hyperglycemic affect [20]. Halia Bara is a traditional health-promoting herb widely used for the treatment of indigestion, sore throats, rheumatism, and hypertension [21]. It has been demonstrated to have various pharmacological activities, such as antiemetic, antiulcer, anti-inflammatory, antioxidant, antiplatelet, and anticancer activities [22, 23]. Furthermore, it has antimicrobial potential as well which can help in treating infectious diseases.

The therapeutic properties of these herbal plants have led us to investigate its anti-protozoal activity against in-vitro growth of *Blastocystis* sp. The herbal extract with the highest efficacy was further purified and its effects were evaluated in comparison with allopathic drugs such as MTZ, Tinidazole, TMP-SMX, Ketoconazole and Nitazoxanide.

## Methods

### Isolation and cultivation of *Blastocystis* sp.

Human-derived isolates of *Blastocystis* sp. were obtained from stool samples of infected individuals. These isolates were cultured in Jones’ medium [24] supplemented with 10 % horse serum and incubated at 37 °C. Subsequently, these isolated cultures were maintained by sub-culturing in fresh Jones’ medium every 3–4 days for at least 1 month prior to the phenotypic analysis.

### Subtyping of *Blastocystis* sp.

*Blastocystis* sp. isolates cultures in Jones’ medium were harvested by centrifugation at 1000 g for 5 min and washed twice using sterile phosphate buffered saline (PBS) (pH 7.4) for the DNA extraction process. The DNA extraction was carried out according to the manufacturer’s protocol using the QIAamp DNA Stool Mini Kit (Qiagen, Australia). The concentration and purity of the DNA was measured using Nanodrop 2000 (Thermo Scientific, USA). 5 μl of DNA preparations was used to amplify the genomic sequences in 25 μl of PCR mixture. PCR amplification for each primer pair was repeated three times for each isolate. Isolates were then subjected to sequenced tagged site (STS) primer PCR using seven sets of STS primers (SB83, SB155, SB227, SB332, SB340, SB336 and SB337).

### Preparation of herbal crude aqueous extracts

Dried Misai Kucing was purchased from a plantation in Juaseh, Negeri Sembilan and leaves of Mas Cotek were purchased from a local plantation in Rembau, Negeri Sembilan, Malaysia while the dried roots of Tongkat Ali, rhizome of Halia Bara and leaves of Putat were purchased from a local plantation in Sungai Buloh, Selangor, Malaysia. Plant samples were deposited in the Herbarium, Rimba Ilmu, University of Malaya, Malaysia and issued with voucher specimen number (KLU46468, KLU46470, KLU47215 and KLU48175). The dried plant parts were ground into coarse powder and subjected to aqueous extraction procedure following the method of Misbah *et al.* [25]. Approximately 100 g of each material was immersed into 1 L of distilled water and boiled for 2 h. At the end of the 2 h boiling period, another portion of 1 L distilled water was added to the boiling solution and extraction process was continued subsequently for 2 h. Once cooled, the extracts were filtered, centrifuged and freeze-dried resulting in lyophilized crude aqueous extract and stored at 4 °C until used.

### Preparation of water and ethyl acetate fraction of crude aqueous extract

The crude aqueous extract that exhibited the highest efficacy against *Blastocystis* sp. in the screening process was further extracted for its water and ethyl acetate fraction to test against these isolates. Briefly, at a ratio of 1:3, 100 ml of the crude aqueous extract was partitioned with 300 ml of ethyl acetate (Merck, Darmstadt, Germany) in a separating funnel. The solution was left still for 15 min to allow the solvent-water separation and thereafter the 2 separated layers, bottom being the aqueous layer and the top being the ethyl acetate layer were collected in two separate glass containers. Subsequently, the aqueous layer was again partitioned with 300 ml of fresh ethyl acetate for another 15 min. The subsequent layers obtained were pooled together with the earlier fractionation step. This process was repeated twice. The aqueous layer was freeze dried resulting in the water fraction while the ethyl acetate layer was evaporated under reduced pressure using a rotary evaporator (Buchi) until a viscous mass was formed resulting in ethyl acetate fraction and stored at 4 °C until used.

### Drug susceptibility assays

Growth profile studies were carried out in order to evaluate the anti-protozoal property and efficacy of crude herbal extracts against *Blastocystis* sp. Being the predominant subtype, ST3 isolate of *Blastocystis* sp. was used in this screening process. The isolate was also tested with MTZ (Sigma-Aldrich, USA), being the current choice of drug as a reference. The screening process was carried out with two concentrations, 0.1 mg/ml and 1.0 mg/ml. A 10 mg/ml stock solution of the crude aqueous herbal extracts was prepared. Allopathic drugs were prepared by dissolving 0.1 g of the respective materials into 1 ml of 0.1 % DMSO (Merck, Darmstadt, Germany) and then serially diluted to obtain the required final concentrations. This assay was carried out in 1 ml micro-centrifuge culture tubes. Briefly, each culture tube contained a parasite count of 10 x 10^4^ viable cells that were pooled together from day 3 of culture while in log phase. 100 μl of the desired concentration of extracts and drugs were introduced into the culture tubes and thereafter fresh Jones’ medium containing 10 % of horse serum was added into the culture tube to achieve a final volume of 1 ml and incubated at 37 °C. A culture tube containing only the parasite of the same volume with fresh Jones’ medium supplemented with 10 % horse serum and 100 μl of 0.1 % of DMSO solvent was prepared as control. Prior to this study, the effect of 0.1 % of DMSO solvent against *Blastocystis* sp. was assessed and the results demonstrated that the susceptibility of the parasite was not affected by the solvent.

This experiment was carried out in triplicates. After 72 h of incubation, the efficacy of the herbal extracts and drugs against this protozoan were assessed by the viable cell count method. This was done by diluting 10 μl of the cell sample in 0.5 % Trypan blue solution at a ratio of 1:1. Viable cells remained unstained whereas the non-viable cells were stained blue. A haemocytometer chamber (Improved Neubauer, Hausser Scientific) was used to carry out the cell count.

### Assessing influence of subtypes on treatment

The herbal extract with the highest efficacy and comparable results to MTZ was further fractionized into water and ethyl acetate fractions. Growth profile studies with the purified fractions were carried across ST1, ST3 and ST5 at 0.1 mg/ml and 1.0 mg/ml. Along with this, allopathic drugs MTZ, Tinidazole, TMP-SMX, Ketoconazole and Nitazoxanide (Sigma-Aldrich, USA) that are commonly associated as first and second line treatment, were also evaluated for its response across ST1, ST3 and ST5 at the same concentrations.

### Compound analysis using Liquid Chromatography - Tandem Mass Spectrometry (LCMS/MS)

LC-MS/MS analysis of both water and ethyl acetate fraction was conducted using AB Sciex 3200QTrap LC-MS/MS with Perkin Elmer FX 15 uHPLC system, equipped with an Agilent Zorbax C18 column (150 mm × 4.6 mm × 5 μm). Solvent A (water with 0.1 % formic acid and 5 mM ammonium formate) and solvent B (acetonitrile with 0.1 % formic acid and 5 mM ammonium formate) were used as mobile phases, and the gradient run program was set as follows: 10 % to 90 % B from 0.01 to 80 min, held for 3 min, return to 10 % B in 0.1 min and re-equilibrated for 5 min. The electron spray ionization was operated in positive mode, with a scan range of 100–1200 m/z for full scan and 50–1200 m/z for MS/MS scan. Prior to injection into the column, both fractions were diluted up to 500 ppm and filtered with 0.22 μm nylon filter. A volume of 20 μl sample was injected for analysis. Mass fragmentations were based on published journal references and ACD/Labs advanced chemometrics mass fragmentation predictive software.

### Statistical analysis

ANOVA test was used to confirm the statistical significance of the results using SPSS version 19. The results were expressed in terms of mean ± standard deviation. All data presented are mean values of triplicate measurements (*n* = 3).

## Results

### Screening of crude herbal extracts against *Blastocystis* sp. ST3

**Fig. 1:**
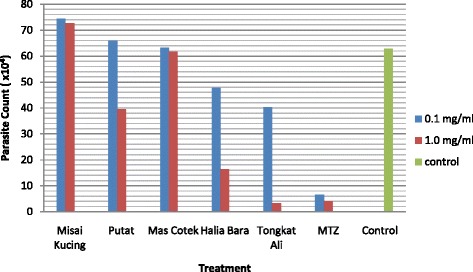
Efficacy of crude herbal extracts against *Blastocystis* sp. ST3. Parasite count after 72 h upon treatment at concentrations 0.1 mg/ml and 1.0 mg/ml. MTZ was used as the reference drug

Growth profile studies were carried out in order to evaluate the anti-protozoal property and efficacy of crude herbal extracts against *Blastocystis* sp. Based on Fig. 1, at 72 h, the parasites in the control culture tubes reached its peak, with a viable cell count of 62.80 × 10^4^ cells whereas when treated with 0.1 mg/ml of Tongkat Ali, Halia bara and MTZ during the same period, the parasite numbers were 40.266 × 10^4^, 47.866 × 10^4^ and 6.66 × 10^4^ cells respectively. In contrary, exposure to Misai Kucing, Putat and Mas Cotek at 0.1 mg/ml demonstrated no effect on the growth of *Blastocystis* sp. cells. When cultures were treated with an increased concentration of 1.0 mg/ml, Tongkat Ali showed the highest decline in viable cell count to 3.33 × 10^4^ cells followed by MTZ (4.13 × 10^4^ cells), Halia Bara (16.40 × 10^4^ cells), Putat (39.60 × 10^4^ cells) and Mas cotek (61.60 × 10^4^ cells).

### Assessing influence of subtypes on treatment

**Fig. 2:**
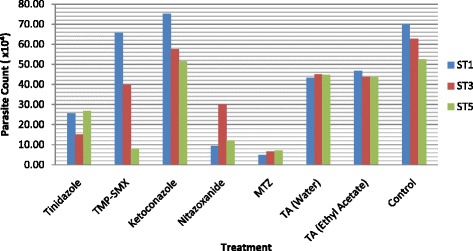
The response of *Blastocystis* sp. isolates ST1, ST3 and ST5 upon treatment. Parasite count after 72 h upon treatment at 0.1 mg/ml

**Fig. 3:**
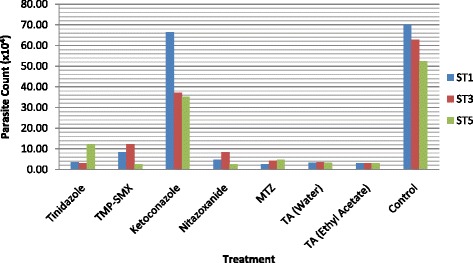
The response of *Blastocystis* sp. isolates ST1, ST3 and ST5 upon treatment. Parasite count after 72 h upon treatment at 1.0 mg/ml

Growth profile studies were carried out in order to study the influence of ST1, ST3 and ST5 of *Blastocystis* sp. when treated with allopathic drugs in comparison to Tongkat Ali (TA) water and ethyl acetate fractions at 0.1 mg/ml and 1.0 mg/ml. Figures 2 and 3 show the responses of isolates upon treatment after 72 h. At 1.0 mg/ml, Tinidazole exhibited higher sensitivity towards ST1 and ST3 isolates in comparison to ST5 isolate. TMP-SMX however expressed great variation in the response across subtypes, while not effective towards ST1 isolate at 0.1 mg/ml, showed 84.4 % of growth inhibition towards ST5 and 36.5 % towards ST3 at the same concentration. At 1.0 mg/ml however, TMP-SMX was more effective towards ST1 (86.2 %) instead of ST3 (80.4 %). Ketoconazole was ineffective towards ST1 isolate at both concentrations while exhibited minimum effect across ST3 and ST5 in comparison to the other treatments. Nitazoxanide showed highest efficacy against ST1 (84.6 %) isolate at 0.1 mg/ml however, at an increased concentration, ST5 (95.1 %) was more sensitive towards the treatment. Amongst all the treatments at 0.1 mg/ml, MTZ exhibited the highest growth inhibition across all three subtypes ST1 (91.9 %), ST3 (89.3 %) and ST5 (86.4 %). At 1.0 mg/ml, the percentage of growth inhibition continued to increase across ST1 (95.8 %), ST3 (93.4 %) and ST5 (90.8 %). Tongkat Ali fractions when exposed to *Blastocystis* sp. isolates at 0.1 mg/ml showed minimum growth inhibition, however at 1.0 mg/ml, both fractions showed high anti-protozoal properties. The water fraction demonstrated growth inhibition of 94.5 %, 94.4 % and 93.6 % across ST1, ST3 and ST5 isolates respectively while the ethyl acetate fraction also exhibited similar percentage of growth inhibition across ST1 (94.9 %), ST3 (95.1 %) and ST5 (94.3 %).

### Identification of chemical constituents in active fractions of Tongkat Ali

LCMS/MS analysis done on water and ethyl acetate fractions of Tongkat Ali extract, exhibited the presence of several possible compounds majorly from the quassinoid and alkaloid class. A total of 10 compounds were identified from the water fraction; six quassinoids, three β-carboline alkaloids and one canthin-6-one alkaloid. As for the ethyl acetate fraction, 9 compounds were identified: seven quassinoids, one β-carboline alkaloids and one canthin-6-one alkaloid. The identified compounds are listed in Tables 1 and 2.

**Table 1:** Composition of potential compounds in the water fraction of *E.longifolia* using LCMS/MS

Class	Identified compound	RT (min)	Chemical formula
Quassinoids	13 β,18-Dihydroeurycomanol	1.71	C_20_H_28_O_9_
	13,21-Dihydroeurycomanone	3.3	C_20_H_26_O_9_
	16-α-o-Methylneoquassin	3.1	C_15_H_24_O_4_
	3,4-Dihydrochaparrinone^a^	3.75	C_19_H_24_O_9_
	Eurycomalactone	5.9	C_19_H_24_O_6_
	Laurycolactone B^a^	6.5	C_18_H_20_O_5_
Canthin-6-one alkaloids	Canthin-6-one^a^	7.45	C_14_H_8_N_2_O
β-carboline alkaloids	1-Methoxymethyl-beta-carboline	4.75	C_13_H_12_N_2_O_3_
	7-hydroxy-beta-carboline 1-propionic acid	4.4	C_14_H_12_N_2_O_3_
	β-Carboline-1-propionic acid^a^	4.5	C_14_H_12_N_2_O_2_

^a^Compounds found in both water and ethyl acetate fractions

**Table 2:** Composition of potential compounds in the ethyl acetate fraction of *E.longifolia* using LCMS/MS

Class	Identified compound	RT (min)	Chemical formula
Quassinoids	3,4-Dihydrochaparrinone^a^	3.6	C_19_H_24_O_9_
	(α/β-epoxide) Ailanthone	3.8	C_20_H_24_O_8_
	Eurycomalide A	6.1	C_19_H_26_O_6_
	Eurycomalide B	5.69	C_19_H_24_O_6_
	Eurycomanol	3.4	C_20_H_26_O_9_
	Eurycomanone	4.0	C_20_H_24_O_9_
	Laurycolactone B^a^	6.4	C_18_H_20_O_5_
Canthin-6-one alkaloids	Canthin-6-one^a^	7.35	C_14_H_8_N_2_O
β-carboline alkaloids	β-Carboline-1-propionic acid^a^	4.4	C_14_H_12_N_2_O_2_

^a^Compounds found in both water and ethyl acetate fractions

## Discussion

*Blastocystis* sp. is the most commonly found organism in any stool survey [26, 27] causing diarrhea, cramping abdominal pain, and nonspecific gastrointestinal symptoms such as flatulence and nausea [28, 29]. Based on the screening process, it was clearly established that amongst all the crude extracts, Tongkat Ali exhibited the highest percentage of growth inhibition and the results were comparable to the reference drug, MTZ. Tongkat Ali is known for its abundant bioactive constituents that contain mostly alkaloids and quassinoids [15]. Over the years, pharmacological evaluations on the various compounds obtained from this plant showed that Tongkat Ali exhibited anti‐malarial, anti‐tumor, anti-bacterial properties. Previously, studies by Taiwanese scientists showed that isolated compounds from the roots of Tongkat Ali exhibited “strong cytotoxicity” towards human lung and breast cancer cell lines [30, 31]. Another study reported 10 new quassinoids from this plant with cytotoxicity effect towards the highly metastatic HT-1080 human fibrosarcoma cell line [32]. Based on a study carried out to evaluate the anti-bacterial activity of Tongkat Ali, it was reported that 1 ml of aqueous leaf extract with a concentration of 100 mg/ml, inhibited the growth of *Staphylococcus aureus* by 82.8 % [13].

This study is the first to report the anti-protozoal property of Tongkat Ali in its crude form against *Blastocystis* sp. isolates. This study has also attempted to fractionate purer portions of the crude extract. The anti-protozoal property of this extract was further evaluated by fractionizing this extract into water and ethyl acetate fractions. These fractions were tested against *Blastocystis* sp. isolates across ST1, ST3 and ST5 and based on the data, both these fractions demonstrated similar effects in terms of the percentage of growth inhibition of *Blastocytstis* sp. isolates and the results were comparable to that of MTZ, the reference drug. This observation suggested that there is a common active compound in both the fractions that is expressing the anti-protozoal property. These fractions were then furthered for the analysis of potential active principles using the combined method of chromatography and tandem mass spectrometry, the liquid chromatography mass spectrometry (LCMS/MS). This analysis revealed that both fractions contained several types of quassinoids, β-carboline alkaloids and Canthin-6-one alkaloids (Tables 1 and 2). Four compounds were present in both fractions; 3,4-Dihydrochaparrinone, Laurycolactone B, β-Carboline-1-propionic acid, and Canthin-6-one. These compounds have previously been proven to possess therapeutic properties. Antiprotozoal activity of Laurycolactone B had been shown and reported previously [33]. Canthin-6-one from Tongkat Ali was reported to show antimalarial as well as cytotoxic effect against human lung cancer (A-549) and human breast cancer (MCF-7) cell lines [15]. Other canthin-6-one isolated from *Zanthoxylum chiloperone* stem bark had been reported to be helpful in the relief of burden due to *Leishmania amazonensis* infection in BALB/c mice [34]. Another study investigated the anti-parasitic effect of canthin-6-one in BALB/c mice infected acutely with *Trypanosoma cruzi*. Based on the data, the parasiteamia was significantly reduced following oral treatment with canthin-6-one [35]. β-Carboline-1-propionic acid has been known to acquire significant anti-malarial activity against cultured *Plasmodium falciparum* strains [36].

Previous studies have suggested that *Blastocystis* sp. isolates of different ST groups express varying susceptibility towards treatment [37, 38]. The variation in responses when treated with chemotherapeutic drugs has made it far from straightforward to eradicate this protozoan. The present study also included the evaluation of the responses of different ST groups of *Blastocystis* sp. when exposed to Tongkat Ali fractions in comparison to allopathic drugs such as MTZ, Tinidazole, TMP-SMX, Ketoconazole and Nitazoxanide. The study showed that there is great variability in responses across ST when exposed to the allopathic drugs. However in the case of the Tongkat Ali fractions, at 1.0 mg/ml, the sensitivity of the isolates across ST showed more uniformity indicating that these fractions not only expressed comparable results to the current choice of drug, MTZ but also shows less variation in susceptibility across ST1, ST3 and ST5.

## Conclusion

Based on the outcome of this study, it is vital to further investigate in depth the mechanism of action and the correlation between subtypes and variations in drug susceptibility. The genotype of *Blastocystis* sp. is highly polymorphic and therefore genomic analysis of ST is essential in order to explicate the differences in pathogenicity, virulence and also in understanding the variation in responses towards treatment. This study shows preliminary potential of Tongkat Ali as anti-protozoal agent however further research such as evaluating Tongkat Ali extracts from various geographical location, further isolation and identification of active principles of the plant extract responsible for the anti-protozoal activity is required in order to develop future pharmaceuticals.

## Ethical approval

This study was approved by the Medical Ethics Committee of the University Malaya Medical Centre (UMMC) (Kuala Lumpur, Malaysia) according to the Declaration of Helsinki approved this study (Reference Number: 848.28).
